# An Investigation of the JAZ Family and the CwMYC2-like Protein to Reveal Their Regulation Roles in the MeJA-Induced Biosynthesis of β-Elemene in *Curcuma wenyujin*

**DOI:** 10.3390/ijms241915004

**Published:** 2023-10-09

**Authors:** Yuyang Liu, Shiyi Wu, Kaer Lan, Qian Wang, Tingyu Ye, Huanan Jin, Tianyuan Hu, Tian Xie, Qiuhui Wei, Xiaopu Yin

**Affiliations:** 1 School of Pharmacy, Hangzhou Normal University, Hangzhou 311121, China; yuyangliu123@163.com (Y.L.); wushiyi0429@163.com (S.W.); lan93kaer@163.com (K.L.); 13865043605@163.com (Q.W.); yetingyu666@126.com (T.Y.); huananjin@hznu.edu.cn (H.J.); hutianyuan007@126.com (T.H.); xbs@hznu.edu.cn (T.X.); 2 Key Laboratory of Elemene Class Anti-Cancer Chinese Medicines, Engineering Laboratory of Development and Application of Traditional Chinese Medicines, Collaborative Innovation Center of Traditional Chinese Medicines of Zhejiang Province, Hangzhou Normal University, Hangzhou 311121, China

**Keywords:** *Curcuma wenyujin*, jasmonate ZIM-domain, MYC2-like protein, transcriptional regulation, β-elemene

## Abstract

β-Elemene (C_15_H_24_), a sesquiterpenoid compound isolated from the volatile oil of *Curcuma wenyujin*, has been proven to be effective for multiple cancers and is widely used in clinical treatment. Unfortunately, the β-elemene content in *C. wenyujin* is very low, which cannot meet market demands. Our previous research showed that methyl jasmonate (MeJA) induced the accumulation of β-elemene in *C. wenyujin*. However, the regulatory mechanism is unclear. In this study, 20 jasmonate ZIM-domain (JAZ) proteins in *C. wenyujin* were identified, which are the core regulatory factors of the JA signaling pathway. Then, the conservative domains, motifs composition, and evolutionary relationships of CwJAZs were analyzed comprehensively and systematically. The interaction analysis indicated that CwJAZs can form homodimers or heterodimers. Fifteen out of twenty *CwJAZs* were significantly induced via MeJA treatment. As the master switch of the JA signaling pathway, the CwMYC2-like protein has also been identified and demonstrated to interact with CwJAZ2/3/4/5/7/15/17/20. Further research found that the overexpression of the *CwMYC2*-*like* gene increased the accumulation of β-elemene in *C. wenyujin* leaves. Simultaneously, the expressions of *HMGR*, *HMGS*, *DXS*, *DXR*, *MCT*, *HDS*, *HDR*, and *FPPS* related to β-elemene biosynthesis were also up-regulated by the CwMYC2-like protein. These results indicate that CwJAZs and the CwMYC2-like protein respond to the JA signal to regulate the biosynthesis of β-elemene in *C. wenyujin*.

## 1. Introduction

*Curcuma wenyujin* Y.H. Chen et C. Ling (*C. wenyujin*) is a species of *Curcuma* genus in the zingiberaceae family, which is primarily cultivated in Wenzhou City, Zhejiang Province, China. β-Elemene (C_15_H_24_) is a sesquiterpene from the volatile oil of *C. wenyujin* [1], which is an important and effective anti-tumor component due to its broad-spectrum antitumor activity, its inhibition ability of tumor cell migration, and its minor side effects [2,3]. Although plenty of work has been undertaken to increase the content of β-elemene in the cell factory of *Saccharomyces cerevisiae* and *Escherichia coli* [4,5], the *C. wenyujin* plant is still the main source of β-elemene so far. In plants, β-elemene derives from the intermediate products isopentenyl pyrophosphate (IPP) and the double-bond isomer dimethylallyl pyrophosphate (DMAPP), which are respectively biosynthesized through acetyl coenzyme A via the mevalonate (MVA) pathway, as well as glyceraldehyde 3-phosphate and pyruvate via the 2-*C*-methyl-d-erythritol 4-phosphate (MEP) pathway [6,7]. Then, farnesyl pyrophosphate (FPP) is obtained by the condensation of IPP and DMAPP with farnesyl pyrophosphate synthase (FPPS), which is a common precursor for the synthesis of sesquiterpene. Finally, germacrene A is formed by germacrene A synthase (GAS) with the substrate FPP, which is spontaneously converted to β-elemene via cope rearrangement [8,9]. Unfortunately, GAS has not been discovered until now in *C. wenyujin*.

Jasmonates (JAs), including jasmonic acid (JA) and the relational derivatives methyl *cis*-jasmonate (MeJA) and (+)-7-*iso*-jasmonoyl-l-isoleucine (JA-Ile), are momentous signal molecules in plants. JAs induce the biosynthesis of secondary metabolites by triggering transcriptional reprogramming in plants [10]. For example, MeJA induced artemisinin accumulation in *Artemisia annua* and tanshinone increment in *Salvia miltiorrhiza* hairy roots [11,12]. In plants, the JA signal transduction is fully elucidated through decades of research. The protein jasmonate ZIM-domain (JAZ) is the critical negative regulator in JA signal transduction [13]. In normal growth conditions, the concentration of bioactive JAs, namely JA-Ile, is relatively low. At this time, the activation of transcription factors is inhibited by JAZs, thereby inhibiting the expression of JA-responsive genes [14]. When plants are subjected to adverse environments, bioactive JA-Ile increases and binds to its receptors CORONATINE INSENSITIVE1 (COI1) [15]. JA-Ile promotes the binding of Skp1-Cul1-F-box (SCF)^COI1^ to JAZ proteins, followed by ubiquitination and degradation in 26S proteasome. Subsequently, the transcription factors suppressed by JAZ, such as the master switch MYC2 and other factors, are disinhibited and finally activate the expression of relational genes [13,16,17].

JAZ proteins are the core repressors in JA signal transduction, which contain TIFY and Jas as the conserved domains. The highly conserved TIFY motif (TIF [F/Y] XG) is located in the center of JAZ near its N-terminal region. The TIFY domain has been confirmed to be necessary and sufficient for the homomeric or heteromeric interactions of JAZ proteins [18]. The Jas domain at the C-terminus of JAZ, also called the CCT_2 domain, contains an S-L-X(2)-F-X(2)-K-R-X(2)-R core sequence. JAZ interacts with transcription factors via the Jas domain and inhibits their transcriptional activation activity. The LPIARR motif in the N-terminal of the Jas domain is essential for JA-dependent interaction between JAZ and COI1 [19]. The C-terminal of the Jas domain contains the conserved nuclear localization signal (NLS) motif. Moreover, the N-terminal cryptic MYC-interaction domain (CMID) was discovered in some JAZ splice variants, such as JAZ10, JAZ1, JAZ2, JAZ5, and JAZ6 in *Arabidopsis thaliana* [20,21]. The CMID domain can bind with MYC proteins while not forming complexes with JA-Ile and SCF^COI1^. Therefore, JAZs containing CMID domains might participate in the desensitization of JA signaling and the reconstruction of signal homeostasis [20,21]. Numerous studies have shown that JAZ proteins are involved in the regulation of plant secondary metabolism. For example, AaJAZ8 repressed the activation ability of the AaTCP14-AaORA complex for the expression of *DBR2*, which is integral for artemisinin biosynthesis [22]; AaJAZ8 also interacted with MYC2 and may suppress artemisinin biosynthesis [22]; and AaJAZ9 repressed the activity of AaWRKY9 for the transcriptional activation of *AaDBR2* and *AaGSW1*, which positively regulates artemisinin biosynthesis [23]. 

Of those transcription factors targeted by JAZ proteins, MYC2 is considered as the master regulator [24,25], which belongs to basic helix-loop-helix (bHLH)-type transcription factors and typically binds to E-box sequences (CANNTG) within the target promoters, such as the G-box (CACGTG) [26]. MYC2 affected the expression of a variety of JA-responsive genes, thus regulating diverse JA-dependent functions in *Arabidopsis* [27,28]. It was reported that MYC2 could modulate the biosynthesis of terpenoid in plants. For instance, AtMYC2 up-regulated the expression of *TPS21* and *TPS11* to increase the emission of sesquiterpene in *Arabidopsis* inflorescences [29]. AaMYC2 activated *AaCYP71AV1* and *AaDBR2* expression by interacting with their G-box, thereby increasing artemisinin accumulation in *A. annua* [30]. SmMYC2a and SmMYC2b interacted with SmJAZ1 and SmJAZ2 to improve the production of tanshinones and salvianolic acid B in *S. miltiorrhiza* [31,32].

In our previous research, MeJA triggered the gene expression for endogenous JA biosynthesis, JA signal transduction, and terpenes biosynthesis, and then increased the accumulation of β-elemene in *C. wenyujin* [33]. However, the detailed molecular regulation mechanism is not clear yet. In this study, JAZs, the core regulatory factors of the JA signal pathway, were identified and analyzed systematically including their conserved domain, motif composition, and evolutionary relationship in *C. wenyujin*. The differential expression profiles of *JAZ* genes responding to MeJA were also detected. Meanwhile, the interaction of the CwMYC2-like protein with CwJAZs and the transcriptional activation activity of the CwMYC2-like protein were proved. Finally, the function of the CwMYC2-like protein in regulating β-elemene biosynthesis was characterized. These findings uncovered the regulation mechanism of β-elemene biosynthesis induced by MeJA in *C. wenyujin*.

## 2. Results

### 2.1. Identification of the JAZ Family Genes in C. wenyujin

Based on the transcriptome databases of *C. wenyujin*, 20 *CwJAZ*s were identified and named as *CwJAZ1-CwJAZ20*. The sequences of all *CwJAZ*s are listed in Supplementary Table S2. The computed characteristics of the identified CwJAZs including sequences length, molecular weights (Mw), theoretical predicted isoelectric point (pI), and subcellular localization are listed in Table 1. The amino acid sequences length of CwJAZs ranged from 114 (CwJAZ20) to 459 (CwJAZ18). The Mw of CwJAZs ranged from 12.97 kDa (CwJAZ20) to 49.12 kDa (CwJAZ18), and the pI ranged from 5.89 (CwJAZ8) to 10.28 (CwJAZ17). CwJAZs were predicted to be located in the nucleus, chloroplast, mitochondria, or plasma membrane, respectively.

**Figure 1:**
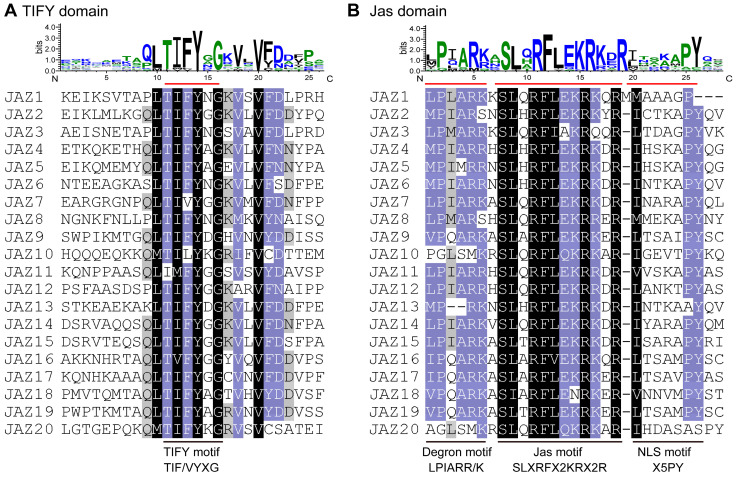
Sequence logos and multiple sequence alignment of TIFY domains (**A**) and Jas domains (**B**) of CwJAZ proteins in *C. wenyujin*. The conserved TIFY/Jas/Degron/NLS motifs were outlined with a red straight line in sequence logos and a black straight line in alignment of the amino acid sequences, respectively. Each stack height indicates the conservation of the sequence at the corresponding position. Each letter height within each stack indicates the relative frequency of the corresponding amino acid. The amino acid residues with a black, purple, and gray background represent 100%, at least 80%, and at least 60% identity, respectively.

The TIFY and Jas domains were highly conserved with some slight variations (Figure 1 and Table 1). Sixteen out of twenty CwJAZs contained the conserved “TIF[F/Y]XG” motif in the TIFY domain. Moreover, there were some variants, such as the “TIVYGG” motif in CwJAZ7, the “TILYKG” motif in CwJAZ10, the “IMFYGG” motif in CwJAZ11, and the “TVFYGG” motif in CwJAZ16 (Figure 1A). The Jas domain of the CwJAZs displayed a highly conserved “SLXRFX2KRX2R” motif, except the “SIARFLENRKER” variant in CwJAZ18 (Figure 1B). The C-terminal of the Jas domain contained the conserved nuclear localization signal (NLS) motif “X5PY”, with some variants such as X5GP, X5AY, and X7PY in CwJAZ1, CwJAZ13, and CwJAZ20, respectively. In addition, the N-terminal region of the Jas domain often contained the degron sequence “LPIARR/K” required for JAZ degradation, although its sequence conservation was not very high. It is worth noting that CwJAZ10 and CwJAZ20 lacked the degron sequence “LPIARR/K”. Perhaps their response mechanism to the JA signal is special [34]. CwJAZ20 contained the most predominant form of the transcriptional repression motif EAR (L × L × L) at the N-terminus (Supplementary Figure S1A), suggesting that it can directly interact with the TOPLESS (TPL) protein without the corepressor NOVEL INTERACTOR OF JAZ (NINJA) [19]. In addition, CwJAZ13 contained both the N-terminal CMID and the Jas domain, which was similar to JAZ10 (At5g13220) in *Arabidopsis* (Supplementary Figure S1B) [20]. CMID had a stronger binding affinity for MYC than the Jas motif of JAZ, suggesting that MYC-JAZ^Jas^ can be replaced by MYC-JAZ^CMID^ [35]. Moreover, ternary MYC–JAZ–COI1 complexes formed, in which Jas mediated the interaction between JAZ and COI1, and CMID mediated the interaction between JAZ and MYC [21].

### 2.2. Phylogenetic Relationships and Motif Composition of JAZs in C. wenyujin

**Figure 2:**
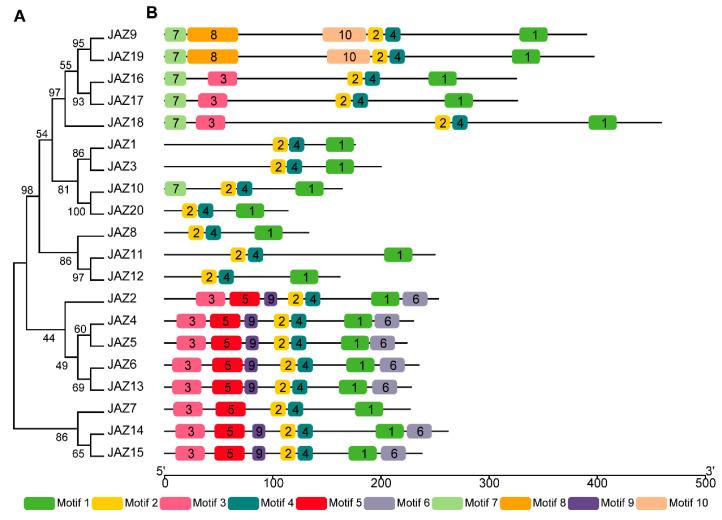
Phylogenetic relationships and motif compositions of JAZ proteins in *C. wenyujin*. (**A**) The phylogenetic tree of CwJAZ proteins was constructed using the neighbor-joining method with a bootstrap of 1000 replicates via MEGA 11. (**B**) Schematic diagrams of motif compositions. Different motifs for CwJAZ proteins were indicated by different colored boxes and numbered 1–10.

The phylogenetic relationships of CwJAZ proteins were analyzed with the software MEGA 11. All CwJAZs were classified into five clusters (Figure 2A). Furthermore, the conserved motifs were identified using the MEME online program to understand the structure characteristics of CwJAZ proteins (Figure 2B). Motif logos are shown in Supplementary Figure S2. All CwJAZ proteins contained motif 1, motif 2, and motif 4, which constituted the conserved TIFY and Jas domains. The motif composition of the variable region outside of the TIFY and Jas domains were disordered, while they were similar in the same evolutionary cluster. These motifs were mainly distributed in the N-terminal of CwJAZs. For example, the CwJAZs in clusters IV and V all contained motif 3/5/9 in the N-terminal and motif 6 in C-terminal, except CwJAZ7. The motifs are related to the function of proteins, but these motifs have not been annotated yet. Moreover, no motif was found in the variable region of CwJAZ1/3/20/8/11/12 except CwJAZ10 in clusters II and III.

### 2.3. Phylogenetic Analysis of JAZ Proteins from Different Plant Species

**Figure 3:**
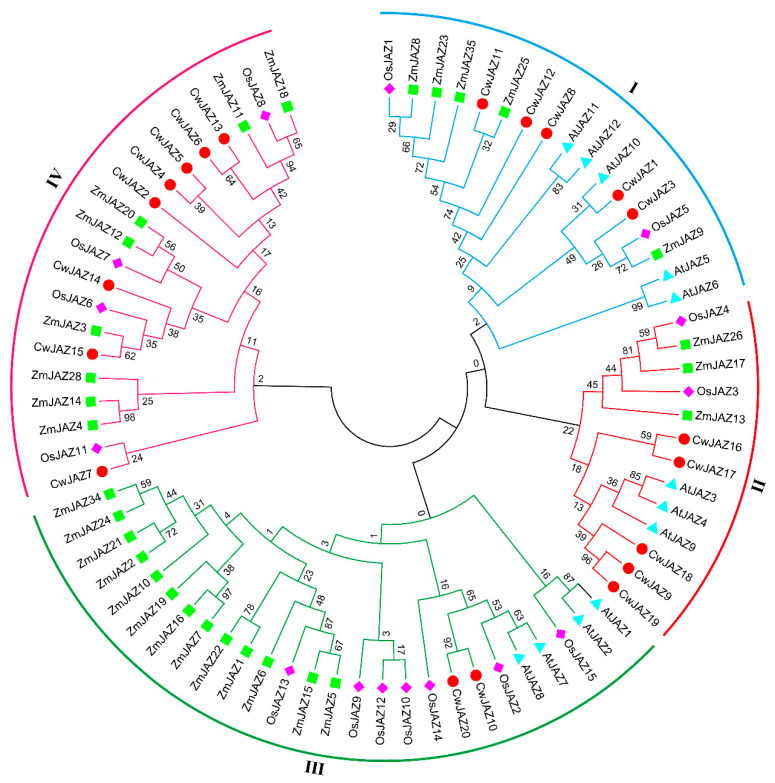
Phylogenetic tree of JAZ proteins from *C. wenyujin*, *A. thaliana*, *O. sativa*, and *Z. mays*. The phylogenetic tree was created using MEGA 11 software coupled with the neighbor-joining method with 1000 bootstrap replicates. Overall, 20 CwJAZs (red circle), 12 AtJAZs (blue triangle), 15 OsJAZs (pink rhombus), and 29 ZmJAZs (green square) were classified into four groups (groups **I**–**IV**).

To understand the evolutionary relationship of the CwJAZ protein family, a neighbor-joining phylogenetic tree was constructed using 76 JAZ proteins from *A. thaliana* of eudicots, *O. sativa*, *Z. mays*, and *C. wenyujin* of monocots (Figure 3). All JAZs were divided into four groups. There was no JAZ of *A. thaliana* presented in group IV, indicating a separation in their evolution. As we all know, homologous genes of different plants may have similar biological functions [36]. For example, AtJAZ5 and AtJAZ10 can restrict COR phytotoxicity and pathogen growth synergistically in *Pst DC3000*-infected leaves [37]. It was speculated that CwJAZ1/3 in subgroup I may play the same roles in *C. wenyujin*. In subgroup II, AtJAZ3 directly interacts with the YABBY (YAB) family transcription factors to attenuate their transcriptional function and repress anthocyanin biosynthesis [38]. AtJAZ4 is involved in plant defense, growth, and development [39]. The results provide a firm reference for the function of homologous genes CwJAZ18/9/19 in subgroup II. In subgroup III, AtJAZ1 attenuates the transcriptional function of WD-repeat/bHLH/MYB complexes, thereby negatively regulating JA-induced anthocyanin accumulation and trichome initiation. OsJAZ9 improves potassium deficiency, cold, and high salt tolerance in rice [40,41,42,43]. Homologous genes CwJAZ10/20 in subgroup III may have similar functions.

### 2.4. Expression Profiles of CwJAZ Genes

The expression of *CwJAZs* in different tissues was analyzed using a quantitative real-time polymerase chain reaction (qRT-PCR) and illustrated using a heatmap (Supplementary Figure S3). The results showed that each *CwJAZ* had a different expression pattern in leaves, flowers, and rhizomes, which were obtained from 3-month-old and 6-month-old *C. wenyujin* plants. The expression levels of *CwJAZ3*, *CwJAZ5*, and *CwJAZ10* were higher in rhizomes than those in leaves and flowers. The majority of *CwJAZs* showed the highest expression in flowers, especially for *CwJAZ17*. The expression patterns of *CwJAZ12* and *CwJAZ20* were similar and lower in all tissues. In addition, the expression levels of *CwJAZ1* and *CwJAZ2* were not detected in all four tissues.

**Figure 4:**
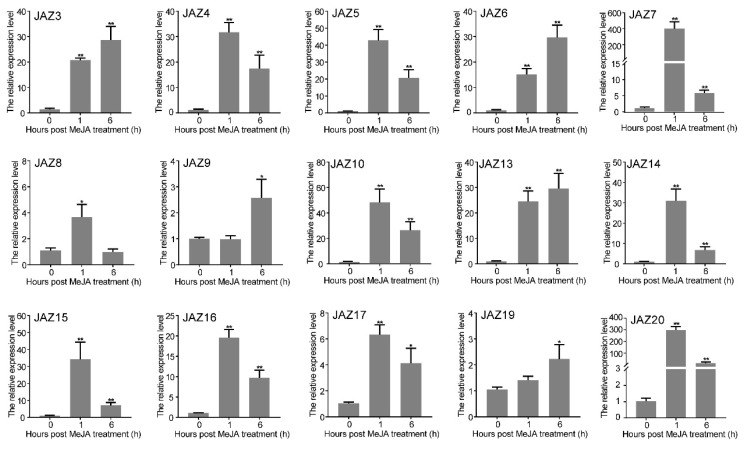
Expression levels of *CwJAZs* under MeJA treatment. Three biological replicates and three technical replicates were performed. Vertical bars refer to ±SE (*n* = 3). Asterisks indicate significant differences (* *p* < 0.05; ** *p* < 0.01).

To investigate the potential roles of CwJAZs in the JA signaling pathway, a qRT-PCR was performed to reveal the expression patterns of *CwJAZs* in *C. wenyujin* leaves with MeJA treatment. The results showed that the expression of 15 *CwJAZs* was significantly induced by MeJA treatment (Figure 4), and 3 *CwJAZs* were hardly induced by MeJA treatment (Supplementary Figure S4). The expression levels of *CwJAZ3*, *CwJAZ6*, and *CwJAZ13* were up-regulated at 1 h and continuously increased to 6 h in *C. wenyujin* after MeJA exposure, while *CwJAZ*9 and *CwJAZ19* were not up-regulated until 6 h after MeJA treatment. The expression levels of *CwJAZ4*, *CwJAZ5*, *CwJAZ7*, *CwJAZ10*, *CwJAZ14*, *CwJAZ15*, *CwJAZ16*, *CwJAZ17*, and *CwJAZ20* were up-regulated at 1 h after MeJA exposure, then decreased at 6 h, which were still higher than those of the control group. The expression level of *CwJAZ8* was up-regulated at 1 h, then restored to approximately control levels at 6 h after MeJA treatment. *CwJAZ1* and *CwJAZ2* could not be detected because of their low expression. 

### 2.5. Interaction between CwJAZ Proteins

**Figure 5:**
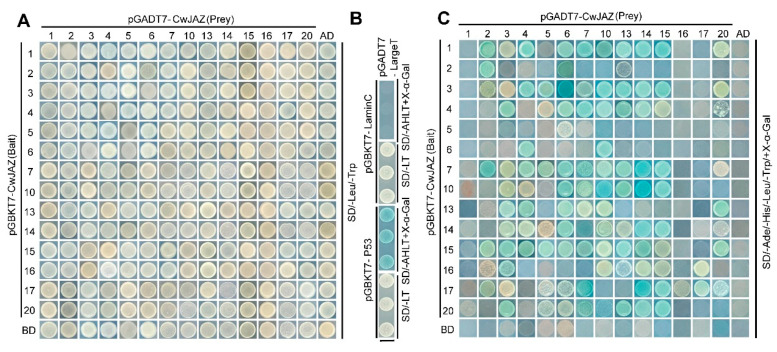
Homo- and heteromeric interactions of 14 CwJAZ proteins via yeast two-hybrid assay. Recombinant plasmids were transformed into yeast strain AH109. Then, co-transformants were screened with SD/-Leu/-Trp medium (**A**) and SD/-Leu/-Trp/-His/-Ade medium with X-α-gal (**C**). (**B**) The co-transformants with pGADT7-LargeT and pGBKT7-P53 were used as positive controls, while those with pGADT7-LargeT and pGBKT7-LaminC were used as negative controls. Three independent experiments were performed.

The dimerization of JAZ proteins may contribute to their stability or promote the simultaneous interaction with MYC proteins [18,44,45]. To reveal the interaction between CwJAZs, a Y2H experiment was performed. The open reading frames (ORF) of *CwJAZs* were respectively cloned into pGBKT7 and pGADT7 vectors to obtain the recombinants GAL4BD-CwJAZ and GAL4AD-CwJAZ. Then, the GAL4BD-CwJAZ and GAL4AD-CwJAZ recombinants were co-transformed into the yeast strain AH109 to determine the interaction. Twelve *CwJAZs* significantly induced by MeJA, and *CwJAZ1* and *CwJAZ2* with low expression levels, were studied. The large T antigen from *Simian virus* 40 has been proved to interact with the P53 protein as a positive control, while it cannot interact with the Lam protein as a negative control (Figure 5B). The positive control, negative control, and 196 combinations grew well on SD/-Leu/-Trp screening medium, indicating that all plasmid combinations were successfully transferred into yeast cells (Figure 5A). At the same time, co-transformants turned blue on SD-Trp/Leu/His/Ade screening medium with X-α-galactoside (X-α-gal), indicating the interaction between CwJAZs (Figure 5C and Table 2). The results showed that seven out of the fourteen CwJAZs including CwJAZ2, CwJAZ3, CwJAZ7, CwJAZ10, CwJAZ14, CwJAZ15, and CwJAZ17 could interact with themselves to form homodimers. Meanwhile, the different hetero-interactions were also observed for all 14 CwJAZ proteins. For instance, 8 CwJAZs containing CwJAZ3, CwJAZ4, CwJAZ7, CwJAZ10, CwJAZ13, CwJAZ14, CwJAZ15, and CwJAZ20 could interact with more than half of 14 JAZ proteins in reciprocal transformations in yeast. Moreover, CwJAZ2, CwJAZ5, and CwJAZ6 interacted extensively with other CwJAZs as the prey rather than the bait. Contrarily, CwJAZ1, CwJAZ16, and CwJAZ17 interacted extensively with other CwJAZs as the bait rather than the prey. All these results indicated that CwJAZ proteins can function in the form of homodimers or heterodimers.

### 2.6. Characterization of CwMYC2-like Protein

**Figure 6:**
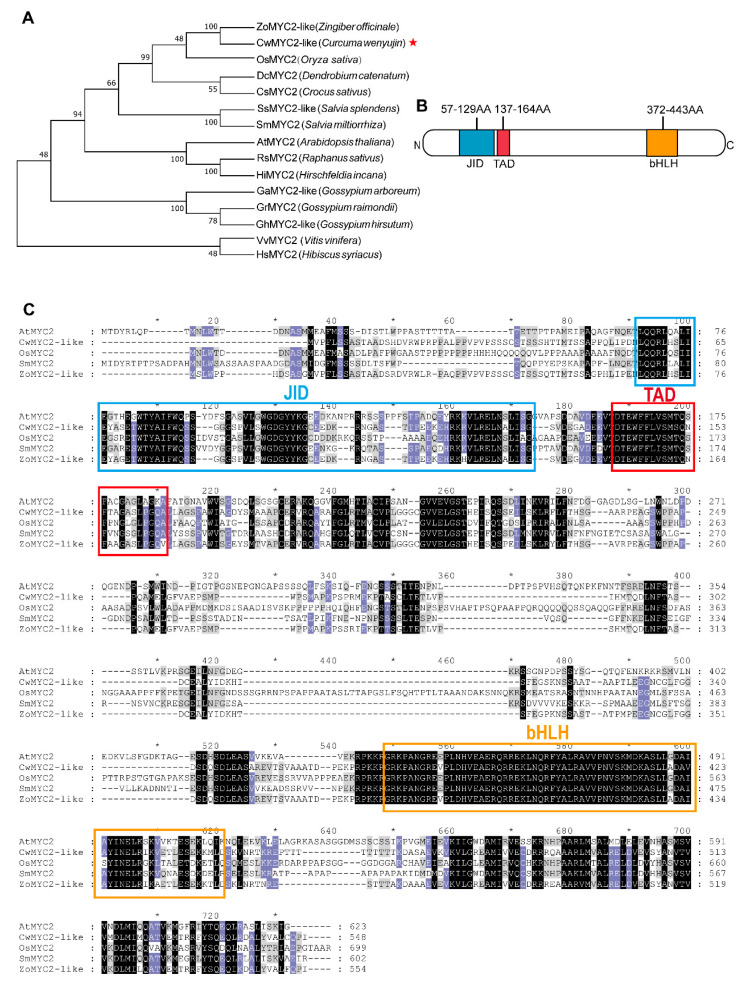
Bioinformatics analysis of the CwMYC2-like protein. (**A**) A phylogenetic tree containing CwMYC2-like and 14 MYC2 proteins in other species. (**B**) Schematic diagram of the CwMYC2-like protein domain. (**C**) Amino acid sequence alignment between the CwMYC2-like protein and other species of MYC2 proteins. The outlined boxes indicate conserved domains. The amino acid residues with a black, purple, and gray background represent 100%, at least 80%, and at least 60% identity, respectively. The asterisk is the marker of an integer.

**Figure 7:**
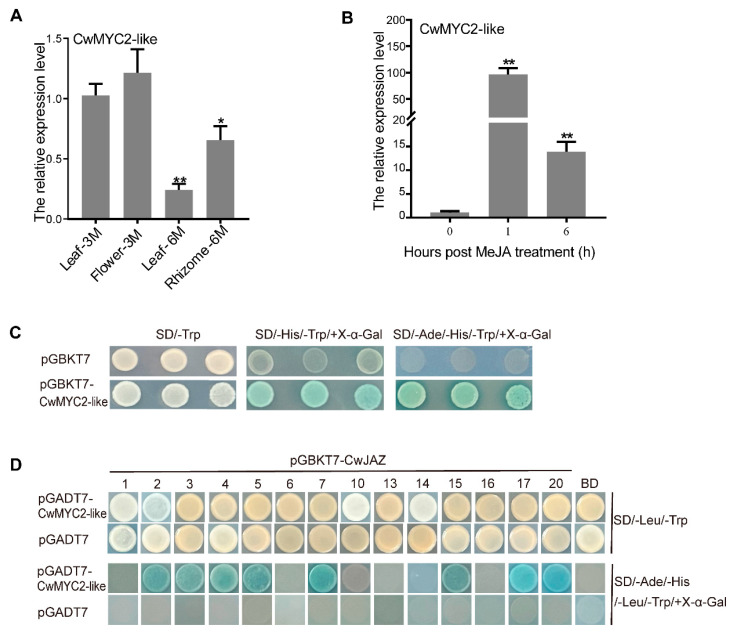
Analysis of expression pattern, transcriptional activation, and interactions of CwMYC2-like protein. (**A**) Expression level of *CwMYC2-like* gene in different tissues of *C. wenyujin*. Leave-3M and Flower-3M: leaves and flower obtained from 3-month-old *C. wenyujin* plant. Leave-6M and Rhizome-6M: leaves and rhizomes obtained from 6-month-old *C. wenyujin* plant. (**B**) Expression levels of *CwMYC2-like* gene in the leaf under MeJA treatment. Three biological replicates and three technical replicates were performed. Vertical bars refer to ±SE (*n* = 3). Asterisks indicate significant differences (* *p* < 0.05; ** *p* < 0.01). (**C**) Analysis of the transactivation activity of CwMYC2-like protein in yeast. Recombinant plasmids were transformed into yeast strain AH109, and then the transformant strains were screened with SD/-Trp, SD/-His/-Trp/+X-a-gal and SD/-Trp/-His/-Ade/+X-a-gal media. (**D**) The interactions between CwJAZ proteins and CwMYC2-like protein. Three independent experiments were performed.

MYC2 is an important switch in JA signal transduction, which is usually inhibited by JAZs when the level of the JA active molecule JA-Ile is low [25]. The *CwMYC2*-*like* protein, a homologous gene to *AtMYC2* (At1g32640) from Arabidopsis, was identified in *C. wenyujin* (Supplementary Table S2). Amino acid alignment results showed that the CwMYC2-like protein is a member of the MYC transcription factor family containing a bHLH domain (PF00010) and a bHLH-MYC_N domain (PF14215) which consists of the JAZ interaction domain (JID) and transcription activation domain (TAD) (Figure 6B,C). The evolutionary relationships of the CwMYC2-like protein with 14 other plant MYC2 proteins showed that the CwMYC2-like protein was highly homologous with the ZoMYC2-like protein in *Zingiber officinale* (Figure 6A). The *CwMYC2-like* gene was expressed in all the test tissues of *C. wenyujin* (Figure 7A). And, the expression level of the *CwMYC2*-*like* gene was significantly up-regulated in the leaf after MeJA treatment (Figure 7B). 

### 2.7. CwMYC2-like Protein Interacted with CwJAZs

To investigate transactivation activity, the ORF of the *CwMYC2*-*like* gene was cloned into a pGBKT7 vector to obtain the recombinant GAL4BD-CwMYC2-like. Then, the GAL4BD-CwMYC2-like was transformed into yeast strain AH109. The transformants grew well on SD/-Trp/-His/-Ade/+X-α-gal screening medium and showed X-α-galactosidase activity, but the negative control pGBKT7 did not (Figure 7C). The results indicated that the CwMYC2-like protein had transactivation activity as a transcription factor.

In order to study the interaction between the CwJAZ and CwMYC2-like protein, a Y2H assay was performed (Figure 7D). The GAL4BD-JAZ and GAL4AD-MYC2-like recombinants were co-transformed into AH109 to determine the interaction. The results showed that the CwMYC2-like protein can interact with CwJAZ2/3/4/5/7/15/17/20.

### 2.8. Transient Over-Expression of the CwMYC2-like Gene Enhanced the Accumulation of β-Elemene in C. wenyujin

**Figure 8:**
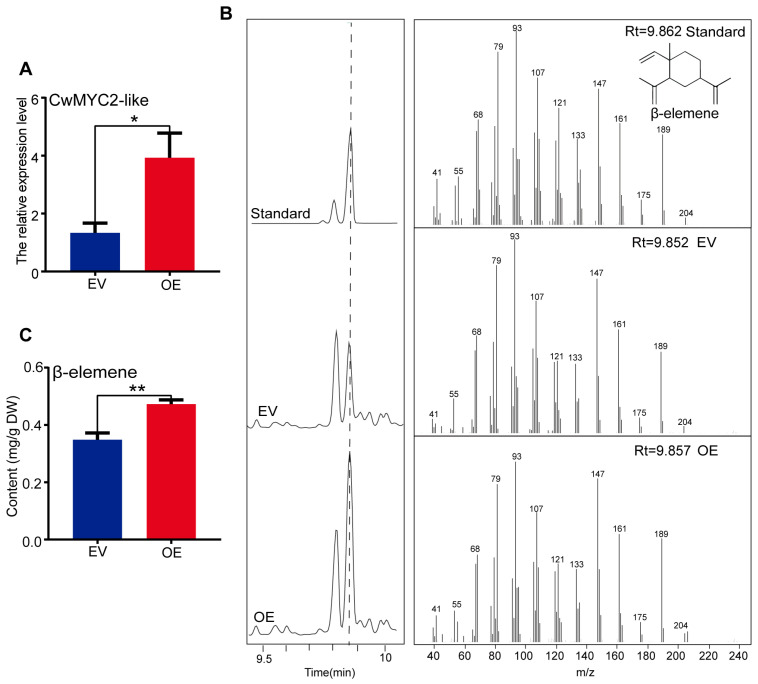
The biological function of CwMYC2-like protein in *C. wenyujin*. (**A**) Expression level of *CwMYC2*-*like* gene in control (EV) and overexpression (OE) lines. (**B**) Gas chromatography–mass spectrometry (GC-MS) analysis of β-elemene content extracted from EV and OE leaves. (**C**) The content of β-elemene in control and OE lines. Three biological replicates were performed. Vertical bars refer to ±SE (*n* = 3). Asterisks indicate significant differences (* *p* < 0.05; ** *p* < 0.01).

**Figure 9:**
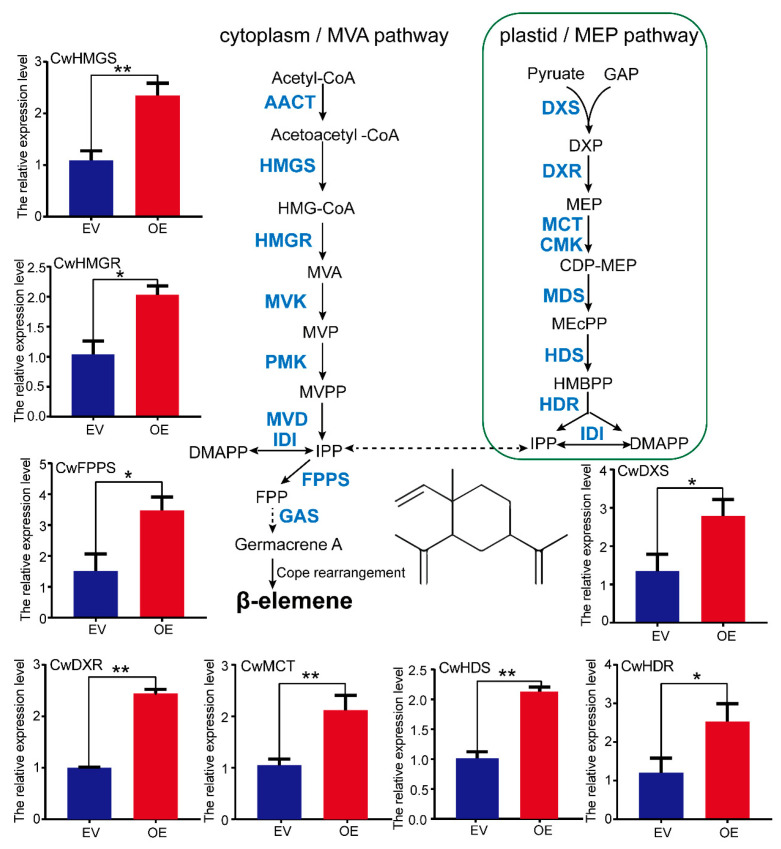
Expression levels of structure genes related to β-elemene biosynthesis. Three biological replicates and three technical replicates were performed. Vertical bars refer to ±SE (*n* = 3). Asterisks indicate significant differences (* *p* < 0.05; ** *p* < 0.01). EV and OE lines represent *A. tumefaciens* GV3101 carrying the pBI121-GUS empty vector and GV3101 carrying the pBI121-CwMYC2-like-GUS recombinant plasmid, respectively. HMGS, 3-hydroxy-3-methylglutaryl-CoA; HMGR, 3-hydroxy-3-methylglutaryl-CoA reductase; DXS, 1-deoxy-d-xylulose 5-phosphate synthase; DXR, 1-deoxy-d-xylulose 5-phosphate reductoisomerase; MCT, 2-*C*-methyl-d-erythritol 4-phosphate cytidylyltransferase; HDS, 1-hydroxy-2-methyl-2-butenyl 4-diphosphate synthase; HDR, 4-hydroxy-3-methylbut-2-enyldiphosphatereductase; FPPS, farnesyl diphosphate.

To investigate the physiological role of the CwMYC2-like protein, the *CwMYC2*-*like* gene (pBI121-CwMYC2-like-GUS) driven by the CaMV35S promoter was conducted in the leaves of *C. wenyujin* via *A. tumefaciens* GV3101-mediated transformation. The GUS staining results indicated that the fusion protein CwMYC2-like-GUS was normally expressed in *C. wenyujin* leaves (Supplementary Figure S5). The expression level of the *CwMYC2*-*like* gene in the overexpression line (OE) was significantly increased compared to that in the control lines with the empty vector (EV) (Figure 8A). β-Elemene, the target ingredient of *C. wenyujin*, was qualitatively analyzed using gas chromatography–mass spectrometry (GC–MS) (Figure 8B). The overexpression of the *CwMYC2-like* gene resulted in a 1.36-fold increase compared to the EV line (Figure 8C). The expression patterns were further detected for the genes related to β-elemene biosynthesis. The expression levels of genes, which encode HMG-CoA synthase (HMGS) and HMG-CoA reductase (HMGR) in the MVA pathway, DXP synthase (DXS), DXP reductoisomerase (DXR), MEP cytidyltransferase (MCT), HMBPP synthase (HDS), and HMBPP reductase (HDR) in the MEP pathway, were all up-regulated in the OE lines compared with the EV lines. In addition, the expression level of the *farnesyl pyrophosphate synthase* gene (*FPPS*), encoding a key enzyme to catalyze the synthesis of the β-elemene precursor, was also increased in the OE lines compared with the EV lines (Figure 9). These results suggested that the CwMYC2-like protein plays a positive regulator role in β-elemene biosynthesis by up-regulating the related gene expression.

## 3. Discussion

β-Elemene, isolated from the essential oil of *C. wenyujin*, is widely used to treat various cancers in clinics [46,47,48]. The content of β-elemene only accounts for 4~10‰ of the dry weight in *C. wenyujin* rhizomes. Although β-elemene can be produced in engineered *Escherichia coli* and *Saccharomyces cerevisiae* via semisynthetic [4,5], the output of these two systems is not enough to meet industrial demand yet. *C. wenyujin* plants are still the most important source of β-elemene. In our previous research, the biosynthesis of β-elemene was significantly increased in *C. wenyujin* with MeJA treatment [33]. However, the specific molecular mechanisms had not yet been elucidated.

The expression of genes involved in endogenous JA-Ile biosynthesis were triggered by MeJA in *C. wenyujin* [33]. Similarly, additional MeJA induced the self-activation of JA biosynthesis in *Hevea brasiliensis* and *Pogostemon cablin* [49,50]. Therefore, the various physiological processes induced by exogenous MeJA in plants are related to JA signal transduction. JAZ proteins are the key regulator factors of the JA signaling pathway [13]. However, JAZs in *C. wenyujin* are entirely unknown to date. Based on RNA-seq data, 20 *CwJAZs* from *C. wenyujin* were identified for the first time (Figure 1; Table 1). In *A. thaliana*, *O. sativa*, and *Z. mays*, 13, 15, and 26 JAZ proteins have been identified, respectively [13,16,51,52]. The lack of *C. wenyujin* genome data may affect the integrity of CwJAZ family identification. In the future, the genome sequencing of *C. wenyujin* will be an important step towards a comprehensive understanding of the CwJAZ family. It is well known that protein function is closely related to structure [53]. Motif composition analysis revealed the identity of motifs in the same cluster, and the differences of motifs between different clusters in non-conservative regions of CwJAZs, indicating the redundancy and diversity of CwJAZs functions (Figure 2; Supplementary Figure S1). However, these motifs are mostly orphan and have not been identified with specific functions yet [54]. In addition, JAZs in model plants such as Arabidopsis, rice, and maize have been widely researched. Homologous proteins in different species may play similar roles. Therefore, the phylogenetic analysis of CwJAZs with JAZs from Arabidopsis, rice, and maize provided a reference for the functional research of JAZs in *C. wenyujin* (Figure 3).

Since JAZ proteins are transcriptional regulators without a putative DNA-binding domain, JAZs interact with other transcription factors to play a regulatory role [55,56]. A high level of JA-Ile enhances the interaction between JAZ and SCF^COI1^, which leads to the degradation of JAZ repressors via the 26S proteasome, resulting in the release of interacting transcription factors, thereby activating the JA-regulated response [13,16,17]. In *C. wenyujin*, MeJA induced more fluctuations for the transcript abundance of 15 *CwJAZ* genes (Figure 4). It was speculated that they may play a role in JA signal transduction. For example, *SmJAZ8*, significantly induced by MeJA treatment, acts as a core transcriptional repressor regulating the JA-induced biosynthesis of salvianolic acids and tanshinones in *S. miltiorrhiza* [57]. SmJAZ1/2/5/6/9 were activators of JA-induced tanshinone biosynthesis and repressors of JA-induced salvianolic acid B biosynthesis [58]. In *C. wenyujin*, 7 CwJAZs (CwJAZ2/3/7/10/14/15/17) formed homodimers, and 14 CwJAZs that formed heterodimers with other CwJAZs were identified (Figure 5). These results are consistent with that in *Arabidopsis* [18], *Prunus persica* [59], *Taxus media* [60], and so on. There is a hypothesis that the homomeric interaction of JAZs may contribute to protein stability, and the heteromeric interaction of JAZs may promote the simultaneous binding of multiple JAZs with MYCs [18,44].

Researches have revealed that JAZ protein regulates plant physiological processes in response to JA signals mainly by interacting with or releasing MYC2 [16,17]. In this study, a *CwMYC2*-*like* gene was identified and cloned, which was significantly induced in *C. wenyujin* by MeJA treatment (Figure 6 and Figure 7A,B). Eight CwJAZs (CwJAZ2/3/4/5/7/15/17/20) were found to interact with the CwMYC2-like protein (Figure 7C,D). Previous research has shown that JAZ proteins are functionally redundant. For example, there were no obvious jasmonate-related phenotypes observed in *Arabidopsis* with a single knockout of *JAZ2, JAZ5, JAZ7*, or *JAZ9* [16]. Therefore, the interaction between different CwJAZs and the CwMYC2-like protein may be functionally redundant in the JA signal response. Of course, it cannot be ruled out that CwJAZs and the CwMYC2-like protein may form multiple regulatory models to participate in the JA signal response. But, further experimental proof is still needed in the future.

MYC2 is considered to be the “master switch” in JA signal transduction [28]. For example, MYC2 in *Arabidopsis* inflorescences integrates both GA and JA signals into the transcriptional regulation of related genes to induce sesquiterpene production [29]. Tobacco MYC2 regulates JA-inducible nicotine biosynthesis genes by binding to the G-box of the target promoters and up-regulating the *NIC2*-locus ethylene response factor (*ERF*) [61]. In *A. annua*, JA-responsive AaMYC2 binds to the G-box of *CYP71AV1* and *DBR2* promoters and activates their expression, thus positively regulating artemisinin biosynthesis [30]. In this study, the transient overexpression of the *CwMYC2*-*like* gene in *C. wenyujin* leaves significantly elevated the accumulation of β-elemene (Figure 8C). β-Elemene is synthesized through the MVA and MEP pathways, which contain a series of catalytic enzymes [7]. HMGS and HMGR are the key enzymes of the MVA pathway [62]. DXS, DXR, and HDR control flux in the MEP pathway [63,64]. FPPS catalyzes substrates to form the precursor of β-elemene. The expression of all these enzyme genes was up-regulated by the CwMYC2-like protein (Figure 9). Taken together, our findings indicated that the CwMYC2-like protein acted as a positive regulator for the biosynthesis of β-elemene by modulating the transcript abundance of related genes.

Based on the above findings, we proposed the working model of CwJAZs and the MYC2-like protein responding to JA signaling for the biosynthesis of β-elemene in *C. wenyujin*. In the absence of JAs, CwJAZs formed homodimers or heterodimers, and then interacted with the CwMYC2-like protein to inhibit its transcriptional activation activity. After the induction of JAs, CwJAZs were degraded and relieved the inhibition of the CwMYC2-like protein. Then, the disinhibited CwMYC2-like protein up-regulated the expression levels of enzyme genes related to β-elemene biosynthesis, thereby increasing biosynthesis and the accumulation of β-elemene. Our study on CwJAZs and the CwMYC2-like protein provides insights for revealing the molecular regulation mechanism of JA-induced β-elemene biosynthesis.

## 4. Materials and Methods

### 4.1. Plant Material and JA Treatment

*C. wenyujin* was obtained from Wenzhou City, Zhejiang Province. Buds of *C. wenyujin* seed tubers were sterilized and then cultured in Murashige and Skoog (MS) solid medium, which was supplemented with 30 g/L sucrose and 3 mg/L 6-benzylaminopurine for the induction of tufted seedlings. One month later, the small tufted seedlings were divided, and then cultured to the trefoil stage with MS solid medium for subsequent JA treatment experiments. The seedlings were cultured in the incubator at 22 °C with a 12 h light/12 h dark photoperiod.

For JA treatment, seedlings with the same development were treated with 250 µM MeJA in MS liquid medium for 1 h to 6 h, respectively. The culture bottles with caps were used to avoid the volatilization of MeJA into the air. Leaf samples with MeJA treatment were collected at 0, 1, and 6 h. For analysis of spatiotemporal expression, the flowers, leaves, and rhizomes of *C. wenyujin* were harvested from Shazhou Village, Taoshan Town, Wenzhou City. Flowers and leaves were collected from 3-month-old plants during the flowering period in June. The rhizomes and leaves were collected from 6-month-old plants during the maturing stage in September. All the samples were frozen immediately with nitrogen and then stored at −80 °C for RNA extraction.

### 4.2. Identification of the JAZ Genes in C. wenyujin

The transcriptome data of different tissues and leaves treated with MeJA from *C. wenyujin* (Accession No. CRA000632, CRA003702, and CRA006461) was available in the Genome Sequence Archive database (https://ngdc.cncb.ac.cn/gsa/, GSA). The Hidden Markov Model (HMM) profiles of the TIFY (Accession No. PF06200) and Jas (Accession No. PF09425) domains were obtained from the Pfam website (http://pfam.xfam.org, accessed on 21 July 2021). The HMM profile was further used as the query to identify CwJAZs in the transcriptome data of *C. wenyujin*, employing TB tools v1.09′ simple HMMER search (E-value ≤ 1×10^−10^). After removing substandard sequences, the conserved domains of identified protein sequences were further confirmed using Pfam 35.0 (http://pfam.xfam.org/search/sequence, accessed on 24 July 2021) and CD-search (https://www.ncbi.nlm.nih.gov/Structure/cdd/wrpsb.cgi, accessed on 24 July 2021) with default parameters. Only those with both TIFY and Jas domains were chosen for further analysis. Multiple amino acid sequence alignment of CwJAZs was performed utilizing the software ClustalW 2.1 and GeneDoc with the default parameters. The sequence logos of conserved TIFY and Jas domains were obtained with the software Weblogo 3 (http://weblogo.berkeley.edu/logo.cgi, accessed on 27 July 2021). Molecular weight (Mw) and theoretical isoelectric point (pI) were predicted utilizing the software “Compute pI/Mw tool” (https://web.expasy.org/compute_pi/, accessed on 27 July 2021). The subcellular localizations of CwJAZs were predicted with the software WOLF PSORT (https://wolfpsort.hgc.jp/, accessed on 27 July 2021).

### 4.3. Phylogenetic Analysis and Conserved Motif Identification

The phylogenetic tree was generated with the software MEGA 11, using the neighbor-joining (NJ) method with 1000 bootstrap replicates [65]. The online program MEME 5.4.1 (http://meme-suite.org/index.html, accessed on 29 July 2021) was used to identify conserved motifs of CwJAZs, with the following empirical parameters: zero or one per sequence, 6–50 amino acids for motif width ranges, and 10 as the maximum number of motifs. Motifs with the E-value of <1 × 10^−2^ were reserved for further analysis. The diagram was completed using TB tools v1.09.

### 4.4. RNA Extraction and qRT-PCR Analysis

Total RNA was extracted with a FastPure Plant Total RNA isolation Kit (RC401, Vazyme, Nanjing, China). First-strand cDNA was obtained with a PrimeScript™RT reagent Kit and gDNA Eraser (Takara, Beijing, China). The LightCycler^®^ 96 System (Roche, Basel, CH) was used for the qRT-PCR utilizing ChamQ Universal SYBR qPCR Master Mix (Vazyme, Nanjing, China). The parameters were set as follows: 60 s at 95 °C, 44 cycles of 5 s at 95 °C, 30 s at 58 °C, and 30 s at 72 °C, and finally 65–95 °C for melting curve detection. The data handling was performed with the comparative 2^−ΔΔCt^ method [66]. The results of the statistical analysis were obtained with the software SPSS v17.0 (IBM, New York, NY, USA). Statistical significance was set as * *p* < 0.05 and ** *p* < 0.01, and 18S rRNA was used as the internal control. The primers used are listed in Supplementary Table S1. The heatmap was generated using TB tools heatmap illustrator [67].

### 4.5. Yeast Two-Hybrid (Y2H) Assay and Transactivation Activity Analysis

The Clontech Matchmaker™ yeast two-hybrid system (TBUSA, State of California, CA, USA) was employed to examine the protein–protein interactions. The ORF of *CwJAZs* and *CwMYC2-like* protein were amplified via PCR and inserted into pGBKT7 or pGADT7 vectors to construct the corresponding recombinant plasmids. Each pair of recombinant plasmids were co-transformed into yeast train AH109, following the manufacturer’s protocol (Takara, Beijing, China) as described before. Then, positive yeast cells were spotted on SD/-Trp/-Leu/-His/-Ade/+X-α-gal solid medium for testing the interaction. pGADT7-largeT and pGBKT7-laminC were used as the negative control; pGADT7-largeT and pGBKT7-P53 were used as the positive control. To analyze the transcriptional activation of CwMYC2-like protein, the ORF of *CwMYC2-like* gene was inserted into the pGBKT7 vector for the recombinant plasmid pGBKT7-CwMYC2-like, which was transformed into yeast train AH109. The plasmid pGBKT7 was used as the negative control. The yeast cells were incubated on screening media at 30 °C for 3 days. The primers used are listed in Supplementary Table S1.

### 4.6. Identification and Transient Overexpression of CwMYC2-like Gene in C. wenyujin Leaves

Protein sequences of MYC2 from *Oryza sativa* and *Arabidopsis* were used to search against the transcriptome data of *C. wenyujin*. Then, the conserved domains bHLH-MYC_N (PF14215) and bHLH (PF00010) of the identified protein sequence were confirmed using Pfam 35.0 and CD-search with default parameters.

The ORF of *CwMYC2-like* gene was cloned into the pBI121 vector, which was driven by the CaMV35S promoter and fused with GUS reporter gene. The pBI121-CwMYC2-like-GUS plasmid was transformed into *A. tumefaciens* strain GV3101 cells. GV3101 cells were cultured until OD_600_ = 0.8 in YEB liquid medium and then resuspended using 1/2 MS liquid medium with 10 mM 2-(N-Morpholino) ethanesulfonic acid (MES), 200 μM adenine sulfate (AS), and 10 mM MgCl_2_. The leaves were collected from *C. wenyujin* seedlings cultured in MS solid medium. The edges and main veins were removed from the leaves, and then cut the leaves into rectangular shapes of a similar size. The rectangular leaf slices were submerged into resuspended GV3101 solution under a vacuum of −0.09 MPa for 10 min, and subsequently suspended at a slower rate for 10 min. After removing the GV3101 liquid from the surface of the leaves, the leaves were cultured in 1/2 MS solid medium with 100 μM AS for 60 h, which were then collected for subsequent gene expression analysis and content detection of β-elemene. The analysis was performed with three biological replicates and three technical replicates.

### 4.7. Extraction and Analysis of β-Elemene

The leaves were dried with a vacuum freeze drier for 3 days. A total of 0.25 g of dried leaf powder was extracted for 12 h with 5 mL n-hexane, and further extracted with 40,000 Hz ultrasonic for 60 min at room temperature. After centrifugation, the supernatant was concentrated to 500 μL with a gentle N_2_ gas flow, which was then filtered through a 0.22 μm organic membrane. The filtrated solutions were then used for qualitative analysis of β-elemene via GC-MS and quantitative analysis via GC. The method referred to our previous research and was briefly described as follows: the initial temperature 80 °C, increased to 140 °C at a rate of 5 °C/min, and raised to 220 °C at a rate of 10 °C/min for 2 min [33]. The method of external standard was used for the quantification.

## Figures and Tables

**Table 1:** Characteristics of identified *CwJAZ*s in *C. wenyujin*.

Gene Name	Protein (aa)	TIFY Motif TIF[F/Y]XG	Jas Motif SLX2FX2KRX2RX5PY	MW (kDa)	pI	Subcellular
*CwJAZ1*	176	TIFYNG	SLX_2_FX_2_KRX_2_RX_5_GP	19.40	9.76	Chloroplast
*CwJAZ2*	253	TIFYGG	canonical	27.61	8.79	Nucleus
*CwJAZ3*	200	TIFYNG	canonical	21.57	9.73	Nucleus
*CwJAZ4*	230	TIFYAG	canonical	25.36	9.60	Nucleus
*CwJAZ5*	224	TIFYAG	canonical	25.15	9.47	Nucleus
*CwJAZ6*	235	TIFYNG	canonical	25.63	9.34	Chloroplast
*CwJAZ7*	227	TIVYGG	canonical	24.99	8.51	Chloroplast
*CwJAZ8*	133	TIFYNG	canonical	14.99	5.89	Nucleus
*CwJAZ9*	390	TIFYDG	canonical	42.07	8.63	Nucleus
*CwJAZ10*	164	TILYKG	canonical	18.73	9.82	Nucleus
*CwJAZ11*	250	IMFYGG	canonical	26.40	9.49	Plasma membrane
*CwJAZ12*	162	TIFYGG	canonical	17.28	9.66	Nucleus
*CwJAZ13*	228	TIFYDG	SLX_2_FX_2_KRX_2_RX_5_AY	24.80	9.24	Chloroplast
*CwJAZ14*	262	TIFYGG	canonical	28.10	8.70	Chloroplast
*CwJAZ15*	238	TIFYGG	canonical	25.75	7.81	Chloroplast
*CwJAZ16*	325	TVFYGG	canonical	35.70	9.63	Nucleus
*CwJAZ17*	326	TIFYGG	canonical	35.41	10.28	Nucleus
*CwJAZ18*	459	TIFYAG	SIX_2_FX_2_NRX_2_RX_5_PY	49.12	9.19	Nucleus
*CwJAZ19*	397	TIFYAG	canonical	43.28	8.88	Nucleus
*CwJAZ20*	114	TIFYKG	SLX_2_FX_2_KRX_2_RX_7_PY	12.97	9.55	Mitochondria

“_” represents the variant amino acid of the TIFY and Jas motif.

**Table 2:** Protein–protein interactions between 14 CwJAZs via yeast two-hybrid assay.

	pGADT7-CwJAZ (Prey)															
pGBKT7-CwJAZ (Bait)		**1**	**2**	**3**	**4**	**5**	**6**	**7**	**10**	**13**	**14**	**15**	**16**	**17**	**20**	**AD**
	1	-	++	+	++	-	++	++	++	++	++	++	-	-	+	-
	2	-	++	-	-	-	-	-	-	+	-	-	-	-	-	-
	3	-	+	+	++	+	++	++	++	++	++	++	-	-	+	-
	4	-	-	++	-	+	++	++	++	++	++	+	-	-	+	-
	5	-	-	-	-	-	+	-	-	-	-	-	-	-	-	-
	6	-	-	-	++	-	-	-	++	-	-	-	-	-	-	-
	7	-	++	+	++	+	++	++	++	++	++	++	-	-	+	-
	10	-	-	+	+	-	++	++	++	++	++	++	-	-	-	-
	13	-	-	++	++	-	++	+	+	-	-	-	-	-	++	-
	14	-	-	+	+	+	++	+	+	+	++	++	-	-	+	-
	15	-	+	++	++	+	++	++	+	++	++	+	-	-	++	-
	16	-	+	++	-	-	-	-	+	+	+	+	-	+	-	-
	17	-	+	+	-	+	+	++	-	-	++	++	+	+	+	-
	20	-	-	++	-	+	+	++	+	++	++	++	-	-	-	-
	BD	-	-	-	-	-	-	-	-	-	-	-	-	-	-	-

“-” represents no interaction, “+” represents weak interaction, “++” represents strong interaction

## Data Availability

The transcriptome data of *C. wenyujin* are openly available in the Genome Sequence Archive (GSA) at http://bigd.big.ac.cn/, reference numbers CRA000632, CRA006461, and CRA003702.

## Supplementary Materials

The following supporting information can be downloaded at: https://www.mdpi.com/article/10.3390/ijms241915004/s1.
