# Supplementary material for: An Investigation of the JAZ Family and the CwMYC2-like Protein to Reveal Their Regulation Roles in the MeJA-Induced Biosynthesis of β-Elemene in Curcuma wenyujin
Source: Int J Mol Sci. 2023 Oct 9;24(19):15004. doi: 10.3390/ijms241915004 (PMC10573570; doi:10.3390/ijms241915004)
Supplement: Supplementary file 1 [file ijms-24-15004-s001.zip › ijms-2630146-supplementary.pdf]

An Investigation of the JAZ family and the CwMYC2-like to reveal their regulation roles in the MeJA-induced biosynthesis of β-elemene in *Curcuma wenyujin*

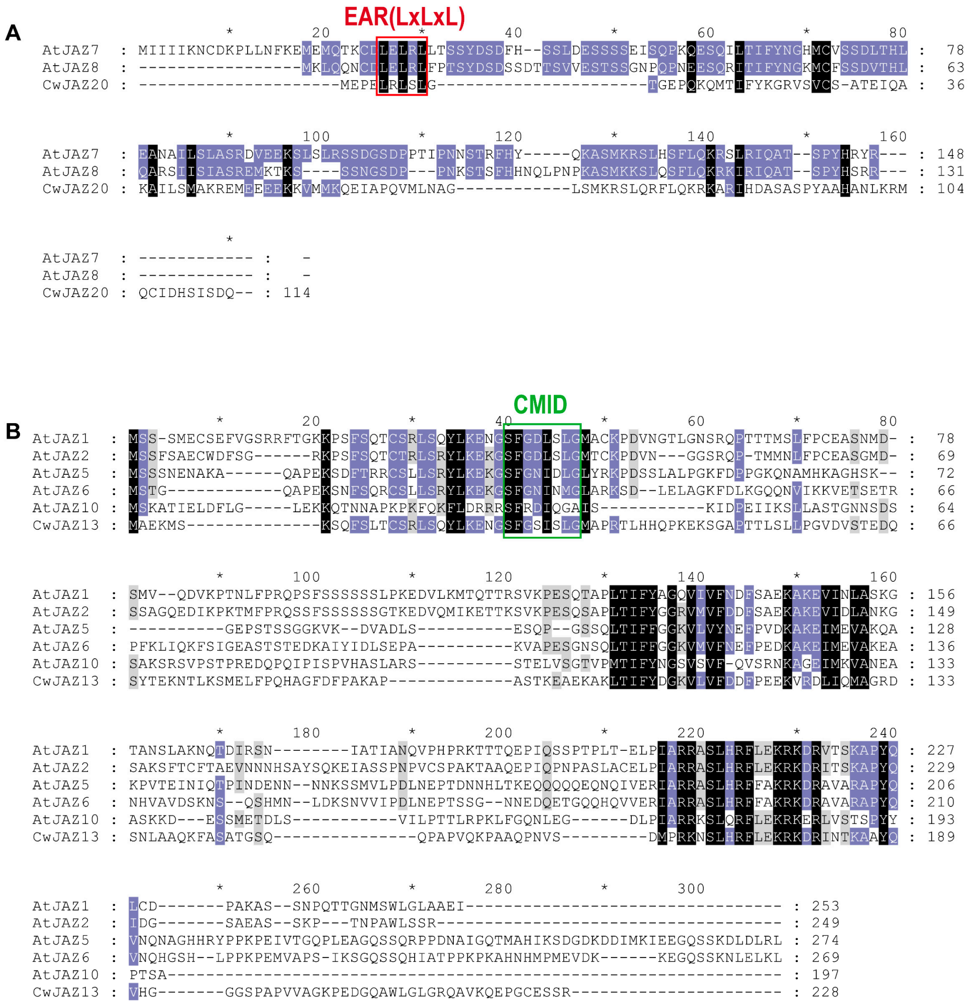

Fig.S1 Special motifs of CwJAZ13 and CwJAZ20.

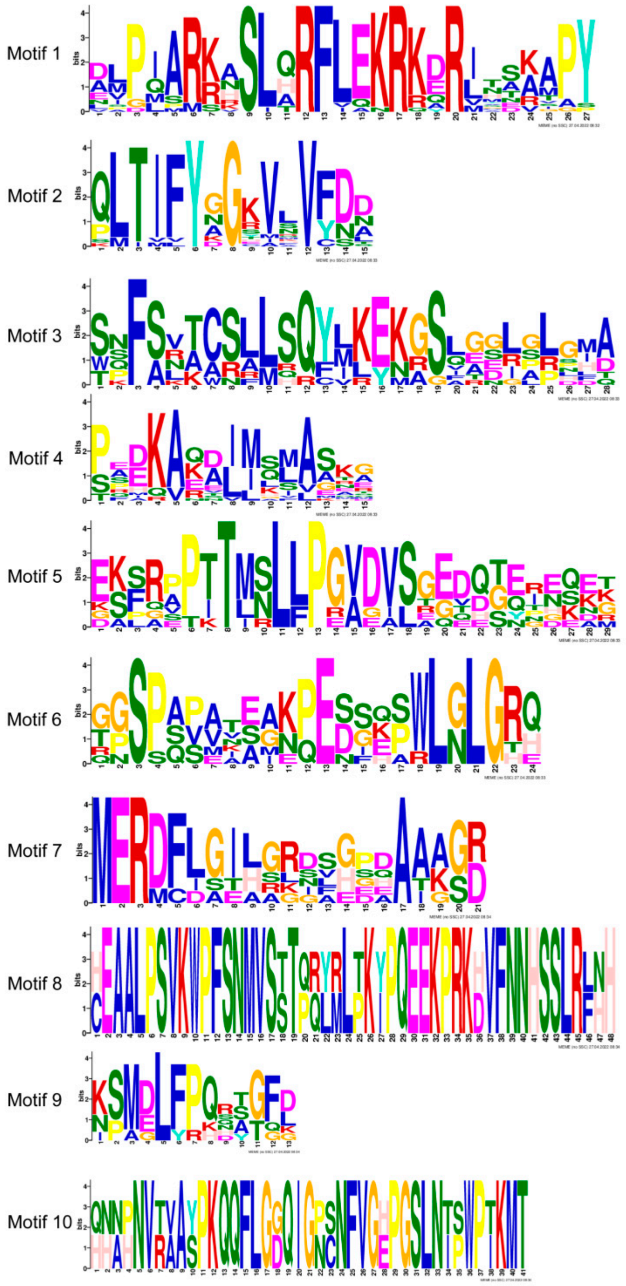

Fig.S2 Sequence logos of motifs 1-10 in JAZ proteins from *C. wenyujin*. Y axis indicates the content of the sequence and its conservation at the corresponding position. The picture was drawn with TB tools software.

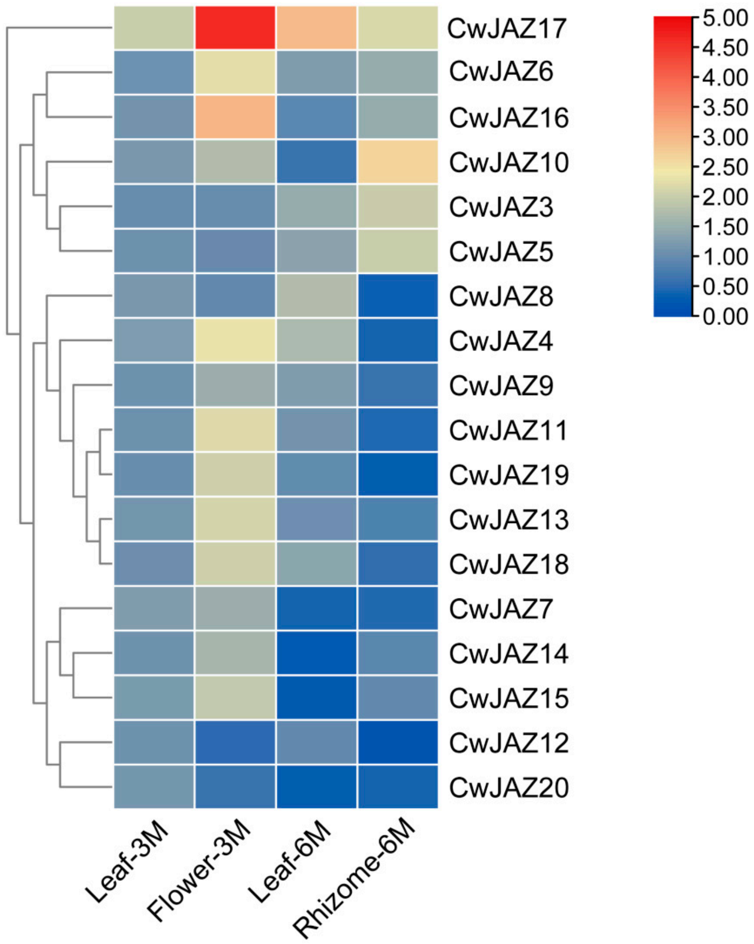

Fig.S3 Expression heatmaps of 20 *CwJAZ* genes in different organizations of *C. wenyujin*. The values were determined via qRT-PCR from three biological replicates. The "Leave-3M, Flower-3M, Leave-6M, Rhizome-6M" heatmaps below indicate leaves, flowers, and rhizomes obtained from 3-month-old and 6-month-old *C. wenyujin* plants, respectively. The expression of "Leave-3M" was considered as the control. All expression levels were calculated using the $2^{-\Delta\Delta Ct}$ method and displayed by the colored boxes. Blue and red represent the two extremes of high and low expression levels.

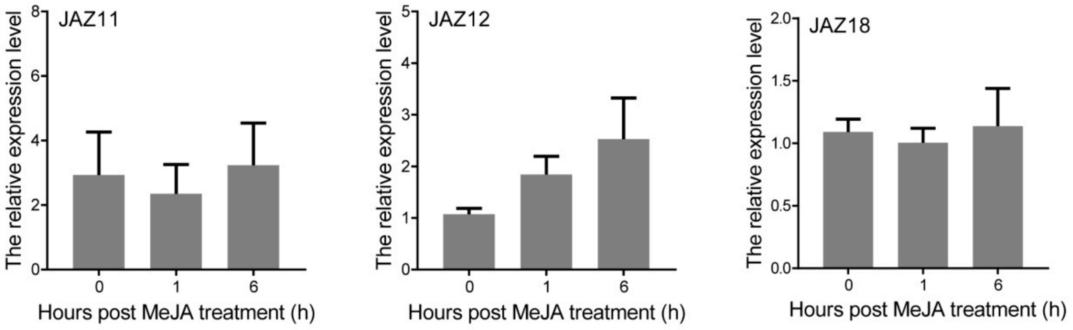

Fig.S4 Expression levels of *CwJAZ* genes under MeJA treatments. Three biological replicates were performed. Vertical bars refer to ±SE (n = 3).

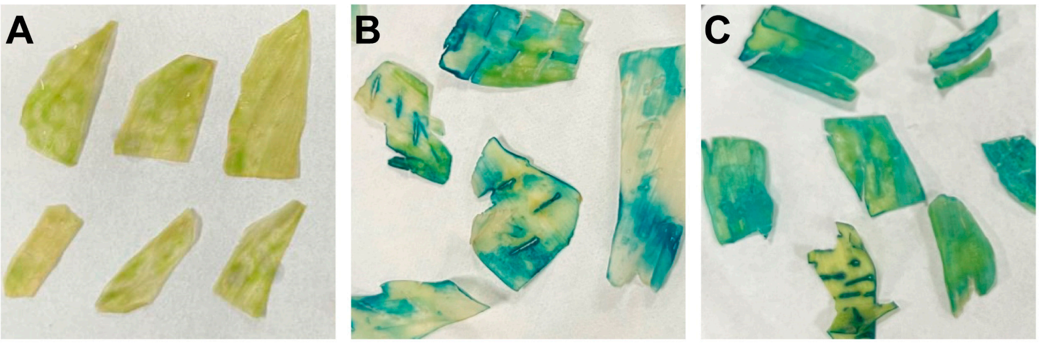

Fig.S5 Gus staining of *C. wenyujin* leaves. (A) Leaves infected with *A.tumefaciens* GV3101. (B) Leaves infected with GV3101 carrying pBI121-GUS empty plasmid. (C) Leaves infected with GV3101 carrying pBI121-CwMYC2-like-GUS recombinant plasmid.
